# Polymer Membranes for Gas Separation

**DOI:** 10.3390/membranes12020207

**Published:** 2022-02-10

**Authors:** Elsa Lasseuguette, Bibiana Comesaña-Gándara

**Affiliations:** 1 School of Engineering, University of Edinburgh, Robert Stevenson Road, Edinburgh EH9 3FB, UK; 2 Institute of Sustainable Processes (ISP), University of Valladolid, 47011 Valladolid, Spain

Over the past decade, polymeric membranes have been widely investigated for a variety of industrial gas separation applications. In today’s competitive and ever-changing environment, membrane gas separation is now widely accepted as an economic process to produce moderate purity stream gases.

This Special Issue on “Polymer Membranes for Gas Separation” of the journal *Membranes* aims to assess the state-of-the-art and future developments in the field of polymeric membranes. Various topics have been discussed, including the synthesis and characterization of novel membrane materials, membrane aging, and the impact of process conditions on transport phenomena, with the desire to improve the gas separation process in all the articles. There are nine contributions, namely, eight articles and one review, in this Special Issue.

Zang et al. [1] synthetized new polymers based on phenylacetylene monomers having one or two carbamate moieties in order to enhance the O_2_/N_2_ separation performance. The presence of two carbamate groups induced a better membrane-forming ability and a higher oxygen solubility and diffusivity thanks to the cistransoid conformation, which is flexible. Thus, these materials presented a greater oxygen permeability compared to the one without carbamate group, 420 Barrer and 3 Barrer, respectively.

In the same way, Rodriguez-Gonzalez et al. [2] synthetized a new set of polyimides in order to study the impact of different chemical moieties (acyclic alkyl-*N*-carbamoyl group with different alkyl chains) on gas properties. For all gases, the authors noticed a decrease in the alkyl chains as they were shortened. These results were correlated with the calculated FFV and the obtained d-spacing values.

Liu et al. [3] described how graphdiyne could make an excellent candidate for hydrogen purification. In their paper, they investigated the gas permeation of four pure gases (H_2_, N_2_, CO_2_, and CH_4_) and binary mixtures through a theoretical study based on MD and DFT calculations. Thanks to its uniform pore size (2.1 Å) and atomic thickness, the graphdiyne presented approximatively infinite selectivities of H_2_ over N_2_, CO_2_, and CH_4_. Moreover, with a slight presence of surface charges, the authors showed that the H_2_ permeance of the binary H_2_/CO_2_ could be increased up to 2 × 8 × 10^5^ GPU, which is several orders of magnitude greater than the existing experiments.

Begni et al. [4] developed new materials to enhance gas separation over time. They synthesized new hypercrosslinked polymers based on the Friedel–Crafts reaction between a tetraphenyl methane monomer and a bromomethyl benzene monomer in order to use them as fillers in PIM-1 mixed matrix membranes. According the reaction process, two fillers have been obtained, with different particle sizes and surface areas, 498 nm and 823 m^2^/g and 120 nm and 990 m^2^/g, respectively. The authors showed that the presence of the fillers induced a slowdown of the membrane’s physical aging, which is greater for the smaller filler. After almost three years of aging, mixed matrix membranes retained approximatively 40% of the initial CO_2_ permeability while pure PIM-1 showed a reduction of 85%. ^13^C spin-lattice relaxation time studies showed that this slow-down in aging is due to the interactions between the PIM-1 chain and the HCP fillers.

The enhancement of gas separation performance could also be achieved by investigating the effect of the process conditions, such as process temperature or the presence of water or other pollutants in the feed. Casado-Coterillo et al. [5] studied the effect of the presence of water and organic pollutants in CO_2_/CH_4_ separation within a hydrophilic and hydrophobic composite membrane. The authors showed that the permeance of the hydrophobic membrane was affected by the presence of damp impurities with an increase in CO_2_ permeance, while the permeance and selectivity of the hydrophilic membrane were almost invariable in the presence of humid streams. By addition of toluene, used as model organic pollutants, the differences were enhanced. Moreover, the CO_2_ permeances decreased with increasing CO_2_ concentration in the feed in a more remarkable way for the hydrophobic PDMS composite membrane than for the hydrophilic IL–CS-based composite membrane, which may be attributed to the water-facilitated transport through the hydrophilic membrane. By consequence, the tuning up of the hydrophilic/hydrophobic character of the membrane surface can be an effective way of improving facilitated transport properties and improving membrane performance in CO_2_ capture applications.

Nemestothy et al. [6] also studied the impact of the presence of pollutants on CO_2_/CH_4_ separation for a polyimide membrane. Commercial hollow fibers based on polyimide have been tested in the presence of naturally occurring contaminants of natural gases, namely, hydrogen sulfide, dodecane, and the mixture of aromatic hydrocarbons (benzene, toluene, xylene). The authors showed that all of the investigated pollutants had an impact on the membrane’s performance but in different ways and to different extents. Hydrogen sulfide increased the permeability of both CO_2_ and CH_4_, and the CO_2_/CH_4_ selectivity had a decreasing tendency as a function of increasing H_2_S exposures. In the case of dodecane, the permeability of CO_2_ and CH_4_ decreased moderately by increasing the degree of exposure, while the CO_2_/CH_4_ selectivity, according to tendencies, was left unaffected. By contrast, the larger exposures of aromatic hydrocarbons caused the increase in gas permeabilities; however, the corresponding trends indicated only marginal changes in the CO_2_/CH_4_ selectivity.

Belaissaoui et al. [7] showed as well that by playing on the selective layer thickness and the carrier concentration of a facilitated transport membrane, it is possible to enhance the separation performance. Their analyses are based on experimental measurements of CO_2_ and N_2_ fluxes through a hybrid fixed-site carrier membrane, based on poly(allyl amine) matrix and on analytical solutions of the facilitation factor mathematically described by means of differential equations expressing a steady-state nonlinear diffusion reaction problem. The dedicated parametric analysis demonstrated that decreasing the selective layer thickness to 0.1 µm together with doubling of the total carrier concentration would theoretically shift the membrane performance far above the Robeson upper bound for the CO_2_/N_2_ pair. However, this potential path for membrane performance improvement has to be weighted by the possible depletion in the reaction complex effective diffusivity.

Benedetti et al. [8] prepared new mixed matrix membranes based on poly(2,6-dimethyl-1,4-phenylene oxide) (PPO) and particles of the size-selective Zeolitic Imidazolate Framework 8 (ZIF-8). The aim was to increase the permselectivity properties of pure PPO. The authors showed that the addition of 45%wt ZIF8 improved the separation performance of PPO by an increase of 800% for CO_2_ and He permeability coefficients. The temperature increase also yielded a simultaneous increase of permeability and selectivity, indicating that such membranes can have potential for applications at high temperatures.

Finally, Vermaak et al. [9] described in their review an overview of membrane-based electrochemical hydrogen separation technologies. Electrochemical membranes are seen as a promising alternative to pressure-driven membranes. Electrochemical membranes are known to generate electricity (fuel cells) or to apply it (water electrolysis), and they are also used to purify/enrich and compress hydrogen streams. They detailed the working principle of electrochemical hydrogen separation and discussed the impact of condition processes, such as temperature, gas mixture, and catalysts, on the separation performance.

In conclusion, the findings and critical discussions from these contributions highlight the importance of membrane materials and the processes for gas separation.
